# Experiences of type 2 diabetes in sub-Saharan Africa: a scoping review

**DOI:** 10.1186/s41256-018-0082-y

**Published:** 2018-09-03

**Authors:** Mikaela Zimmermann, Christopher Bunn, Hazel Namadingo, Cindy M. Gray, John Lwanda

**Affiliations:** 1 Institute of Health and Wellbeing, College of Social Sciences, University of Glasgow, Glasgow, UK; 2 Malawi Epidemiology and Intervention Research Unit, Lilongwe, Malawi

## Abstract

**Background:**

The prevalence of diabetes in Sub-Saharan Africa (SSA) is growing rapidly. Qualitative research on experiences of type 2 diabetes in SSA is emerging, but no qualitative synthesis has been attempted. This scoping review aims to redress this lack of synthesis and to extract policy-relevant suggestions from the literature.

**Methods:**

Scoping review methodology was employed. Eleven online databases were searched (CINAHLplus, Cochrane Library, EBESCOhost, GALE Group, MEDLINE, Pro-Quest, Pscyhinfo, Pubmed, SCOPUS, Web of Science, WorldCat), using terms designed to identify qualitative studies of experiences of diabetes in SSA. Findings from records identified in the search were analysed inductively in NVivo 10 in three stages, to produce an analytical synthesis of studies of diabetes experiences in SSA.

**Results:**

Searches were conducted in 2017 and identified 2743 records, which were reduced to 21 after screening. The earliest identified record was published in 2003 and there was a clustering of records published between 2014 and 2016. The 21 records were based in eight SSA countries: Cameroon, Ethiopia, Ghana, Senegal, South Africa, Tanzania, Uganda, and Zimbabwe. A majority of the studies were conducted in Ghana (5) and South Africa (5), limiting the generalisability of our findings.

The analytical synthesis produced five themes: *identifying type 2 diabetes* (how participants conceptualise and position their illnesses); *hybridity of diabetes care* (how multiple forms of care are often blended and/or pursued concurrently); *impediments, improvisation and diabetes management* (describing challenges faced, how these are responded to and management via diet and physical activity); *sources of support* (who supports participants and how); and *diabetes and HIV/AIDs* (the ways in which the two conditions are sometimes confused and how stigma is often experienced).

**Conclusions:**

The experiences of people with type 2 diabetes in SSA are under-researched across the region, pointing to a gap in knowledge. Interpreting our analytical synthesis, we suggest three priority areas for policy makers and implementers. Firstly, uncertainties relating to access to diabetes treatment need to be reduced. Secondly, more needs to be done to acknowledge and alleviate the economic struggles that those with diabetes face. Finally, high-quality information and education would improve recognition and management of the condition.

## Background

The number of adults estimated to be living with diabetes in sub-Saharan Africa (SSA) in 2017 was 15.5 (9.8–27.8) million, with a regional prevalence of ~ 6%, and associated healthcare costs of USD 3.3 billion. By 2045, this number is expected to increase by 162.5%, with an estimated 40.7 million adults suffering from type 2 diabetes and costs rising to USD 6 billion [1]. The increase in diabetes prevalence in SSA is expected to outpace all other global regions [2]. SSA countries face a number of challenges tackling this growing diabetes burden, including limited health and social care resources and the continued costs of diseases such as HIV/AIDS, malaria and tuberculosis [3]. Estimates suggest that as many as ~ 69% of SSA adults living with diabetes are undiagnosed [1, 4], leading to a high prevalence of diabetes-related complications and in 2017 6% of all deaths were attributed to diabetes [5].

In recent years, 89% of African countries have established non-communicable disease (NCD) centres or departments within their Ministries of Health [6]. Although there is a growing wealth of epidemiological evidence to guide national diabetes strategies, the experiences of those who live with the condition have received less systematic attention Studies of ‘illness experiences’ have a long history in European sociology and social psychology, and have focussed on understanding the subjectivities of people living with illness, the coping strategies that they deploy, and how they experience social structures [7]. Individual studies of type 2 diabetes illness experiences in SSA have drawn on this literature and considered the complex meanings the condition is attributed [8], the techniques through which people pursue self-care [9], and the negotiation of biomedicine [10].

Such emerging literature on type 2 diabetes illness experiences in the SSA region has much to contribute to situated and policy-relevant insights, but maximising impact and utility requires a synthesis of the evidence from individual studies. The aim of our review is to redress the lack of synthesis and to assemble all the relevant peer-reviewed evidence in a systematic and accessible way. Further, we aim to contribute to conversations focussed on how to provision for the increasing numbers of people living with type 2 diabetes in SSA, by making policy-relevant suggestions based on findings from studies that report on the experiences of people with type 2 diabetes.

## Methods

This review a scoping review framework inspired by Arksey and O’Malley and Levac [11, 12] as follows: identifying the research question, identifying relevant studies, selecting studies, charting the data, summarising the results. We describe the review process using these five steps, which were carried out by MZ and overseen by CB through regular meetings and discussion.

### Identification of the research question

Our research question was developed through discussion to focus the review on: a target population (people living in sub-Saharan African); a concept (illness experiences); and health condition (type 2 diabetes) [12]. The final question which guided the review was: *In existing qualitative studies, what is known about the illness experiences of people with type 2 diabetes in Sub-Saharan Africa?*

### Identification of relevant studies

To identify studies, keyword sets were utilized in searches of academic databases. These keyword sets were established through an iterative process (test and refine). Concepts of illness experience identified in the literature were employed in the first keyword set (e.g. ‘biographical disruption’ [13]; ‘narrative reconstruction’ [14]; ‘illness work’ [15]). However, the concept of illness experience proved hard to operationalise, and our searches did not return the records we were expecting, including records identified as relevant in advance of the search. Accordingly, we opted for a broad search strategy and the final keyword set was: (Africa) AND (diabetes) AND (qualitative). Eleven databases were searched (see Table 1). Searches were carried out in February and March 2017, no limitation was put on the publication date of the records, but we restricted the search to peer-reviewed studies written in English. The references of included papers were checked for papers of potential interest. While Arksey and O’Malley suggest that grey literature should be included, pragmatic, time-based restrictions on the work led us to the decision to exclude this literature. Finally, we consulted with two researchers based in SSA who are engaged in diabetes-related work.

**Table 1:** Number of records returned during searches, by database

Database	N of Records
ProQuest	817
EBESCOhost	99
GALE Group	414
MEDLINE	1382
Cochrane Library	1
Web of Science	126
WorldCat	34
Pubmed	97
Psychinfo	14
CINAHLplus	17
SCOPUS	58
Total Records	2814

### Study selection

We used the following inclusion criteria for the title and abstract screening: studies including primary qualitative empirical data on patient experiences of type 2 diabetes; studies based in a SSA country (using the World Bank’s definition of the SSA region [16]). Reviews were excluded, but no review of diabetes experiences was identified. If abstracts indicated a focus on the experiences of others, such as medical professionals or carers, they were also excluded. After the titles had been screened, the same inclusion and exclusion criteria were applied to the abstracts. While the title screen only looked for *potential* evidence of qualitative data, abstracts were reviewed for *specific* reference to *illness experiences*. Following abstract review, full texts were accessed for final screening and data extraction.

### Charting the data and summarizing the results

Our data charting and exploration was guided by Thomas and Harden’s three-stage approach to qualitative data synthesise [17]. The results sections from all records were imported into NVivo 10, analysed inductively and coded line-by-line by [MZ], using codes discussed and agreed with [CB]. We then grouped related codes to create new ‘top line’ codes to describe the groupings, and used these ‘top line’ codes to create analytical themes. We then tabulated the records to make the distributions of themes across the review clear.

## Results

**Fig. 1:**
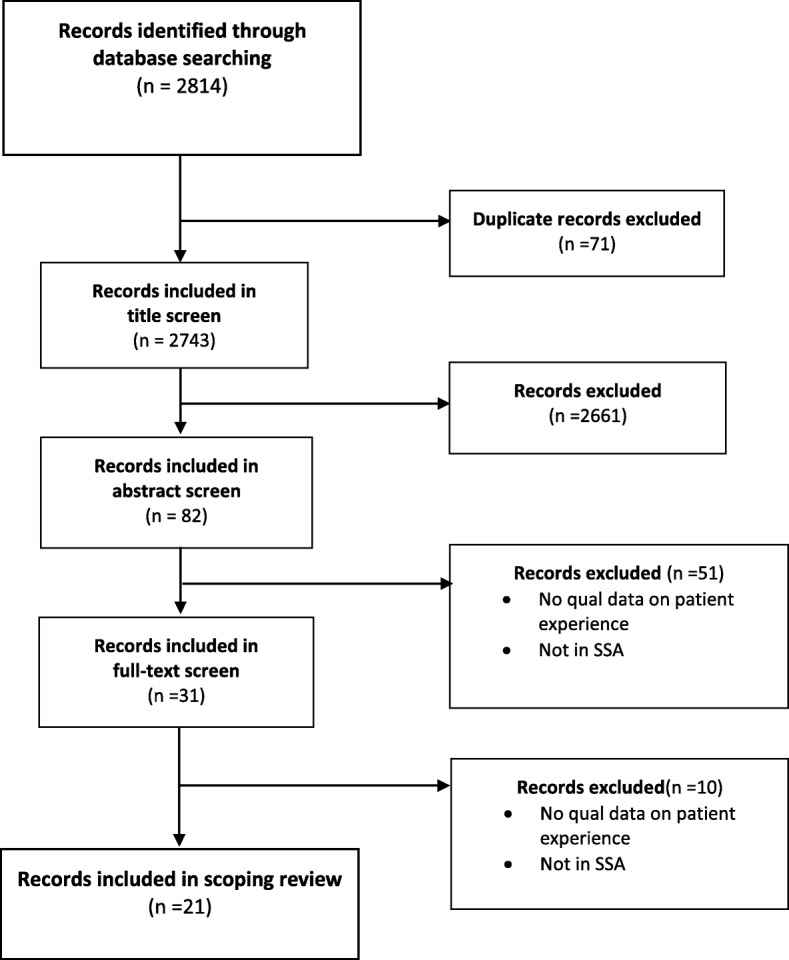
Scoping review flow diagram

**Fig. 2:**
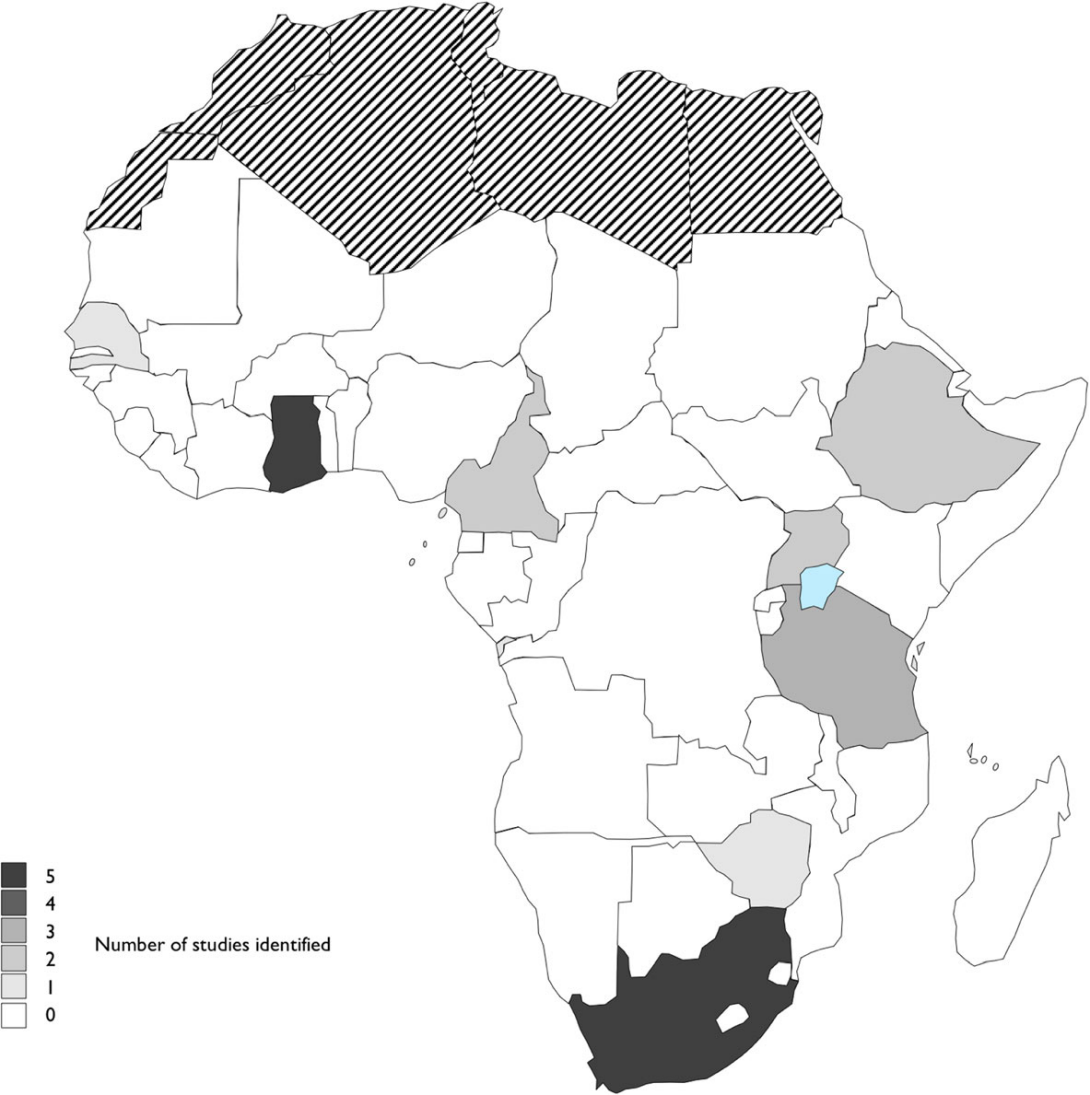
Map of studies of diabetes experiences in SSA identified in this review (countries in black stripes are not part of the SSA region)

**Fig. 3:**
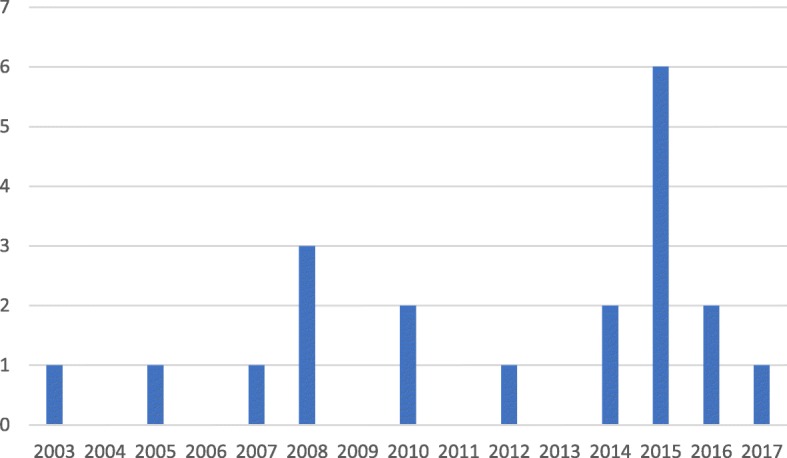
Distribution of studies included in the review by year

The initial search terms yielded 2814 total records (see Table 1). After 71 duplicates were removed, a further 2661 records were screened out during a review of titles. The abstracts screen excluded a further 51 papers, leaving 31 papers to read in full. When screening and reading were completed, 21 papers were included in the final review (see Fig. 1). The 21 articles represented studies in 8 Sub-Saharan countries: Ghana (5), South Africa (5), Tanzania (3), Cameroon (2), Ethiopia (2), Uganda (2), Senegal (1), and Zimbabwe (1) (see Fig. 2 for a map-based visualisation). Of the studies identified, 10/21 were conducted in Ghana and South Africa, and only 8 of 48 SSA countries were represented. Figure 3 graphs the publication dates of the 21 studies and demonstrates the emerging nature of the literature. Median number of participants across the 21 studies was 25, with a range of 9–82 and a total of 594. Table 2 details study methods and participants, as well as a visual representation of themes attributed to each of the 21 papers.

**Table 2:** Studies included in the scoping review

Authors (Year)	Country	Participants	Methods	Themes Covered in the Study				
				Identifying type 2 diabetes	Hybridity of diabetes care	Impediments, improvisation and diabetes management	Sources of Support	Diabetes and HIV/AIDS
Adeniyi et al. (2015) [18]	South Africa	17 people with diabetes with HBA1c ≥ 9% registered in a single clinic	Interviews	x		x	x	
Aikins (2003) [19]	Ghana	28 people with diabetes recruited using snowballing and opportunistic methods	Interviews	x	x	x	x	x
Aikins (2005) [20]	Ghana	26 urban people and 41 rural people with diabetes recruited through 2 clinics, 1 self-help group and snowball sampling	Interviews, Focus group discussion, Ethnography	x	x	x		x
Awah and Philimore (2008) [21]	Cameroon	82 people with diabetes registered at 1 of 4 clinics included in the study	Interview, Ethnography	x	x	x		x
Awah, Unwin and Phillimore (2008) [10]	Cameroon	20 people with diabetes and their families registered at 1 of 4 clinics	Participant Observation, Fieldwork, interviews, Focus group discussion	x	x	x		x
BeLue et al. (2012) [22]	Senegal	54 people with diabetes registered at a single clinic	Interviews	x	x	x	x	x
Booysen and Schlemmer (2015) [23]	South Africa	29 people with diabetes patients with Hgt > 12 mmol/l Recruited through a single community health centre	Interviews, Focus group discussion	x		x	x	
Broder et al. (2014) [24]	Ghana	59 people with diabetes recruited from Ghana’s National Diabetes Research and Management Centre	Focus group discussion	x	x	x		
Doherty et al. (2014) [25]	Ghana	40 people with diabetes registered at a single clinic	Focus groups, semi-structured interviews	x		x		
Habte et al. (2016) [26]	Ethiopia	39 people with diabetes registered at 1 of 3 clinics	Semi-structured interviews	x	x	x		x
Hjelm and Nambozi (2008) [27]	Uganda	25 people with diabetes registered at a single clinic	Semi-structured interviews	x	x	x		
Hjelm and Mufunda (2010) [8]	Zimbabwe	21 people with diabetes registered at a single clinic	Semi-structured interviews	x	x	x	x	
Kagee, Le Roux and Dick, (2007) [28]	South Africa	9 people with diabetes registered at 1 of 4 clinics	Semi-structured interviews	x		x	x	
Mendenhall and Norris (2015) [29]	South Africa	27 people with diabetes recruited from an existing cohort study	Semi-structured interviews	x	x	x	x	
Metta et al. (2015a) [30]	Tanzania	35 people with diabetes recruited from a single clinic	Focus groups, semi-structured interviews	x		x	x	x
Metta et al. (2015b) [31]	Tanzania	19 people with diabetes recruited from the community and the local diabetes clinic	Semi-structured interviews	x	x	x		
Murphy et al. (2010) [9]	South Africa	13 people with diabetes recruited from 1 of 3 community health centres	Semi-structured interviews	x	x	x	x	
Mwangome et al. (2016) [32]	Tanzania	9 people with diabetes recruited from 1 faith-based organisation facility	Semi-structured interviews	x		x	x	x
Nielsen et al. (2016) [33]	Uganda	10 people with diabetes identified through a household survey	Survey, Interviews, Observation	x		x	x	
O’Brien et al. (2015) [35]	South Africa	19 people with diabetes recruited from private and public healthcare providers	Interviews		x	x	x	
Tewahido and Berhane (2017) [34]	Ethiopia	13 people with diabetes recruited from 1 of 2 clinics	Interviews	x		x	x	

Our analytic synthesis produced five themes. *Identifying type 2 diabetes* collects together how participants/informants recognised and interpreted their condition. *Hybridity of diabetes care* reports findings relating to how participants blend approaches (notably biomedical and traditional) to caring for their condition. *Impediments, improvisation and diabetes management* describes findings concerning the challenges that informants reported, how they were overcome, and the steps taken to self-manage through diet and physical activity. *Sources of Support* summarises findings relating to the social, emotional and practical supports that participants reported receiving. Finally, *Diabetes and HIV/AIDS* looks at how informants described the relationships between the two conditions, particularly how the former is often mistaken for the latter.

### Identifying type 2 diabetes

Findings relating to how Participants recognised and interpreted their type 2 diabetes were reported in most papers included in the review [8–10, 18–34]. A recurring observation was that the symptoms of diabetes were often not recognised by those experiencing them, and healthcare professionals tended to be the first to respond to signs of the disease [8, 21, 26, 27, 30, 33]. For example, an informant in Hjelm and Munfunda’s study of health beliefs amongst Zimbabweans with diabetes described:

“...experiencing a feeling of tiredness, loss of weight, dryness of the mouth and passing lots of urine...I got confused because I failed to understand what was happening....feeling of dying...went to seek advice from a sister-in-law (a nurse)...tested my urine...then taken to a private doctor.” [8]

In this instance, a family member who was also a nurse was able to recognise and respond to her brother-in-law’s symptom, after interpreting them as a sign of potential diabetes.

Once diagnosed with type 2 diabetes, many reacted with shock, and some initially perceived the condition as posing an immediate threat to their lives:

“When told that I had this illness I thought I would die the very next day. I didn’t think that I would get to live for all these years because I had no knowledge about the disease.” [26]

However, as this Ethiopian man implicitly narrates, after gaining ‘knowledge’ he understood that the condition need not kill him.

Most studies found that the majority of people with diabetes understood the links between high sugar diets, inactivity, and obesity as contributors to diabetes. However, there was also evidence that some people with diabetes questioned the links between lifestyle and diabetes, particularly when neither their bodies nor lifestyles reflected dominant narratives which positions type 2 diabetes as a disease of affluence [8, 18–20, 22, 25]:

“There is no relationship between obesity and control of diabetes at all, look at me how tiny and poor I am and it is difficult to control my diabetes.” [18]

Echoing this South African woman’s association of diabetes and wealth, one study reported that in Ghana, diabetes is referred to as ‘esikafoo yare’ (‘disease of the wealthy’) [19]. A participant in Senegal develops this narrative further, describing key markers of urbanisation and modernisation as underlying causes of lifestyle transitions which exacerbate diabetes risk:

“the population is so inactive of course diabetes will be very present here. A while ago when we used to be farmers and the population was active we didn’t have diabetes, hypertension or thyroid. With technology cars, computers etc., we became sedentary and we started seeing all of these diseases.” [22]

The arrival of processed food, new technology, and consequent shifts in lifestyle were all offered as drivers of increasing diabetes prevalence.

The chronic nature of diabetes was also a source of contention for study participants [10, 19, 20, 24, 26, 30]. The majority of studies found that people with diabetes understood that diabetes was a chronic condition for which there was no cure, as this Tanzanian woman exemplifies [10, 18, 21, 24, 26, 27, 31, 32]:

“…Diabetes is not a curable disease. Rather, the medicines we are using are just for relief; that is what we were told at the clinic one day.” [31]

Many of these informants were also aware that while diabetes cannot be cured, it can be managed. However, a significant number of studies found that some participants were hopeful that there was a cure. In the studies by Aikins (Ghana) [19], Awah, Unwin and Phillimore (Cameroon) [10], Habte et al. (Ethiopia) [26], and Hjelm and Nambozi (Uganda) [27], some informants rejected the chronic nature of diabetes and instead spoke of a cure for the disease. Awah, Unwin and Phillimore attribute the rejection of diabetes as a chronic condition in part to the belief that some held that all disease should have a cure, suggesting that this motivates searches for understanding and cure in non-biomedical domains [10].

Studies also found that some informants, such as this person from Tanzania, initially attributed their diabetes to witchcraft [8, 10, 19–21, 27, 30]:

“When I contracted diabetes I was very fat. I had a big belly, but it disappeared after only three days. I mean everyone who saw me was surprised, saying, ‘It has only [been] three days!’ That is why I went to the traditional healer. I was afraid because I had changed a lot; it was a big change for just three days. I thought I was bewitched…” [30]

However, witchcraft and appeals to the supernatural were not simple replacements for biomedical explanations. Rather, biomedicine and traditional medicine are not mutually exclusive. [8, 10, 19, 21]:

“I will just finish these[…] drugs because I bought them. But tomorrow, I will go for more protection and tell the native doctor that he is a man. He knows his job. I must not joke at my workplace because there is a lot of witchcraft and jealousy there; they have put this diabetes into my body. My family told me in the village that I should be careful… My man will cure me and that enemy will be ashamed” [21]

As this Cameroonian exemplifies, the two discourses can become entangled.

### Hybridity of diabetes care

Thirteen studies focused on how people with diabetes employed both biomedical and non-biomedical services in the management of their type 2 diabetes [8–10, 19–22, 24, 26, 27, 29, 31, 35]. Employing non-biomedical services was frequently narrated as part of a search for a cure, as described by this Ghanaian [10, 19–21, 30]:

“I want to be healed so I will follow up whenever I hear of somebody who can help.” [19]

Some searches for cures were directly linked to a perceived failure of biomedical practitioners to provide a cure.

“I was never at ease when the doctor told me that I had diabetes. I tried to be cured in the hospital and my diabetes could not finish. The doctor kept telling me that I was not complying with treatment. A friend of mine also, sick with diabetes, told me that some unperformed ritual might be holding back the treatment. She advised me to consult a traditional healer. The healer advised me on the ritual to perform to my ancestors. After the ritual, I felt well and my blood sugar returned to normal six months afterwards. I believe that the doctor did not know this other cause of my diabetes and how I could be cured.” [10]

Informants such as this Cameroonian spoke of frustration at the lack of a cure and continued suffering as reasons for engaging non-biomedical healers. When informants assessed their treatment regimens to be of low efficacy, they looked to other forms of treatment.

The financial pressures that biomedical treatments often place on households was another reason offered for consulting non-biomedical services. Non-biomedical services cost less than biomedical services, providing a cheaper alternative to those struggling to afford to pay for biomedical services, as represented by this Senegalese narrative [10, 19–22]:

“The main problem we have with diabetes is the diet and the medications are extremely expensive. The cheapest drugs are between 60 to 100 dollars a month. For a third world country like Senegal, it is almost impossible for most of us to be able to afford it. Most of the diabetic patients are living in misery or dying. The situation is that extreme. A money issue is the main reason why most of us try traditional plants because it is more economical” [22]

Even though non-biomedical services are routinely utilized, biomedicine was often reported to be both preferred and the first source of diagnosis [9, 19, 20, 22, 24, 26, 35]. An stark example of this comes from South Africa:

“I just follow instructions as the doctor said to me. I decided ok, the doctor told me this and this, which is why I have to follow it, because they know what I don’t know. They saw it in my body…so I just carry on..” [9]

Moreover, when symptoms worsened, non-biomedical explanations and treatments for diabetes tended to be downplayed by participants in favour of biomedical alternatives.

A number of studies described a situation where both biomedical and non-biomedical services were used interchangeably [10, 19, 21, 22, 26, 30]. This mixing of system could be due to the lack of access for biomedical treatments [31], or simply a prevailing notion that mixing traditional and biomedical systems resulted in the better outcomes [10, 19, 20, 24, 26].

‘Let health care providers do their part, the traditional healers will complete the treatment’ [10]

As described by this Cameroonian, hybridising multiple systems is not uncommon. Participants often take multiple forms of medication, mix advice from a variety of healers, and deploy variegated conceptual frameworks in their accounts of how they care for themselves.

Alongside traditional healers and biomedical healthcare professionals, religious beliefs also contribute to treatment discourse. Many informants attributed any potential success in treating their diabetes to God’s will. Those who went to faith-based healers or engaged in prayer as a form treatment considered God to be the ultimate healer [19, 20, 24, 26, 31, 33]. In Cameroon, participants described the belief that God could impact all aspects of treatment: from blessing food and medication, to guiding a person to a good doctor.

“It is only God who can intervene. It is only God who can let you meet a doctor who can cure you of the disease.”

“If God doesn’t bless the drugs you take it might not be effective even though it is efficacious.” [20]

Christian healing manifested itself in many practices including special diabetes prayers, taking communion, being anointed with holy water, and visiting dedicated faith healers. In combination with biomedical services, the inclusion of religion was seen as an effective method to find a cure [8, 19, 20, 24, 26, 27, 29].

### Impediments, improvisation and diabetes management

All 21 studies in the review reported narratives relating to the impediments people with diabetes face when managing their diabetes. The high costs of medication was a major concern for people held by people with diabetes studie [8, 9, 19–22, 27, 28, 31, 32, 34, 35]. As highlighted by BeLue et al., the cheapest medication in Senegal is unaffordable for most [22]. Also patients are often advised to take multiple medications to treat existing or prevent future comorbidities, as narrated in Ethiopia:

“. . .The drugs are not affordable. . .besides diabetes doesn’t come alone. There’s the cholesterol and hypertension that come along. I struggle to cover all that with my government salary.” [34]

Another cost identified in three studies was the cost of diabetes monitoring equipment, with participants in Uganda reporting that managing treatment is made more difficult by an inability to afford monitoring equipment to test their blood sugar levels [8, 18, 27]. “.. . the machine for testing is very expensive. Can’t afford about 60 000 shillings.” [27]

One study reported that informants would sometimes buy their medication directly from local pharmacies without attending clinics to obtain an appropriate prescription, to avoid healthcare and transportation costs [32]. Other methods of managing diabetes under financial constraints were focused on prolonging medication supplies. In Tanzania, this included reducing dosages or sharing medication with other people with diabetes [31, 32].

“…Medicines are very important for treating this disease…That is why you find people using a half of the dose and saving the rest for the days ahead, because you do not know how it is going to be tomorrow… [Have you experienced that situation?] Yes…sometimes instead of injecting myself twice in a day I inject myself only once… At least when I do this the medicine for a month can last me two months.” [31]

Improvisations in treatment practices were not just related to medication, but also occurred as a response to a lack of blood glucose testing equipment. One participant described testing sugar levels by tasting urine for sweetness or looking to see if ants are attracted to it [32].

Accessing clinics, hospitals and other biomedical facilities was a significant impediment for some. In four studies, informants reported that biomedical facilities were too far away to reach easily, often due to the cost of transport. This was especially true for those who lived in remote villages in rural or semi-rural areas [18, 28, 30–32].

“I need to have enough money for a return transport, accommodation, and food before I can think about going to the clinic—and that doesn’t include the money for laboratory tests and medicines… my financial resources are low….I just leave it to God—if I have money I go to get the medicines, and if I don’t, I stay home and go without….” [31]

In addition to the high cost of transportation, participants such as this Tanzanian woman required money for accommodation and food in order to make the journey to her nearest diabetes clinic. During rainy seasons, clinics also become unreachable for some:

“….During the rains it is very difficult to come to the clinic because our roads are impassable, and cars are unable to reach the village….. This is why even I could not come to the clinic last month…” [31]

Even when clinics were accessible, the treatment and care described in the literature was often inadequate [18, 28, 29, 31, 32]. For example, medication was not always available at local health clinics, such as this one in Tanzania:

“…For instance, last year diabetes medications disappeared for almost three weeks … I could not find them anywhere in Ifakara … and during that time I didn’t have a single medicine from the hospital…. That it is when you find us in these other places (local healers/herbalists).” [31]

Alongside disruptions to the supply of medicines, informants also described long delays at health clinics. These delays often entailed losing wages or labour time, and some reported fainting as a consequence of waiting for fasting blood glucose tests [8, 31, 32].

Informants reported how diabetes also impacted their incomes. Six studies recounted the impacts that diabetes had on informants’ ability to work [8, 19, 22, 23, 26, 28, 29]. Diabetes often resulted in early retirement, as a result of diabetes-related impairments.

Complications such as decreased mobility, or poor eyesight were contributing reason for people having to retire early [26]. One Senegalese person simply stated:

“I am blind and cannot support my family.” [22]

Earnings were also lost due to time spent seeking treatment. Kagee, Le Roux and Dick found that participant in South Africa reported losing their wages when they had to go to and travel to biomedical facilities:

“You miss a day’s wages. So you see money plays a role whether or not I go to the clinic. What do you think?” [28]

In some instances, informants described living with diabetes as stressful [8, 9, 21–23, 26, 27, 29, 34], which was often related to financial concerns, as in this Ghanaian case:

“It is frightening when you don’t have money to buy drugs, but if you have money then your mind is at ease.” [19]

As well as financial stresses, participants were concerned about managing their conditions. Specifically, the inability to monitor glucose levels and struggles to keep diabetes under control were both described as sources of stress by South African participants [9, 29]. Finally, family conflicts about food were also sources of stress when the dietary changes to treat diabetes affected household meals [22, 23, 29].

Exercise as a way to manage diabetes was discussed in eleven studies [8, 9, 18, 22–24, 27, 29, 33–35]. There were mixed result in the uptake of exercise. Some informants discussed how they incorporated exercise in daily activities: “I care for the baby so I am always moving around the house” [29]. Reasons for not taking up exercise included pain when exercising, and the view that the environment was not suited to exercise [24, 25, 34, 35]. informants from South Africa said that they did not feel safe enough in the neighbourhoods to exercise outside [23], while participants in Ethiopia lamented the lack of affordable facilities [34].

Another impediment to self-care reported in the literature related to the foods that participantshad access to. Eleven studies focused on how informants found sustaining an appropriate diet a pressure on finances [8, 9, 18–20, 22, 25, 28, 29, 32]. Participants in Senegal described the high cost of vegetables and lean meats [22]. Sourcing sufficient vegetables was a concern for the Informants in Ghana [20]. Others noted that when a type 2 diabetes-appropriate diet was consumed by the entire household, expenditure on food increased [23, 25, 28]. This led one South African to conclude: “No, you will lose your house and your car to afford those foods”. [23]

### Sources of support

Twelve studies reported on the sources of support that people with diabetes were able to draw on [8, 9, 18, 20, 22, 23, 28–30, 32–35]. For many, family and friends were the most important sources of support. Support described came in multiple forms, from providing financial aid to making sure that treatment regimens are followed, as was evidenced by one participant from Senegal:

“I get a lot of support from my family and from my friends thank God. I cannot complain and I am very grateful. My son who was born in 1974 is a grown man and he helps me a lot financially. The rest of my family supports me mentally. My wife and my daughter-in-law cook my food.” [22]

Care informants reported receiving from family often included reminders to take medicines [22, 28, 32, 33], as described by this Ugandan:

“When I’m in bed and my granddaughter has prepared food, she wakes me up and says, ‘Food is ready, take your medicine.” [33]

Metta et al. found that when initially confronted with diabetes symptoms, informants turned to friends (as well as family) for assistance [30]. Like family, friends also provided financial support, by lending cash [8, 31]. A South African informant also described forming supportive friendships with other people in their community who live with diabetes:

“People have been supportive [of my diabetes]. My neighbour now also has it and we are always talking about it together and I enjoy being around her. We usually share a laugh about it sometimes”. [29]

While many participants described living in supportive environments, with helpful family and friends, some narrated feeling unsupported in caring for their diabetes [22, 23, 29, 35]. Three studies found that tensions relating to food often arose in families, including resistance to making changes to household meals to accommodate the diabetes-related needs of one person (South Africa and Senegal) [22, 23, 29]. One Senegalese participant even suggested that her daughter-in-law was acting out of spite towards her:

“...My daughter-in-law knows that I am diabetic but she cooks rice a lot because she doesn’t want me to do well.” [22]

Similarly, tensions over diet could also occur between friends. One study from Ethiopia found that during social events, refusal to eat from a common dish was considered inappropriate, forcing people with diabetes to choose between adhering to their diet or maintaining good relations [34].

Some participants felt that they did not get the necessary levels of support from their health care providers [8, 9, 18, 21, 23, 33, 35]. Booysen and Schlemmer, describe how some participants felt that nurses were too strict with them and humiliated them in front of others [23]. An unsympathetic health care system was also mentioned by participants in Kagee, Le Roux and Dick’s study [28]. While informants in Murphy et al.’s study reported that when doctors voiced disapproval, it made them nervous to ask questions [9]. An additional way in which participants described feeling unsupported by healthcare providers related to the quality of information about their diabetes that was made available [9, 18, 23, 33]. For example, participants in Uganda described how often doctors did not explain what their blood glucose test results were, or failed to provide accessible records:

“At the hospital they don’t tell the numbers—just write them. If they [the nurses] write large I can read them myself, but if they write too small, I have to ask my brother’s granddaughter or the neighbor’s child to read them because I do not see well.” [33]

The lack of education on managing diabetes was mentioned in eight studies [9, 18, 23, 24, 27, 29, 32, 33, 35]. Informants reported that they need more information on medication and diets.

### Diabetes and HIV/AIDS

The HIV/AIDS epidemic in SSA has had an impact on the ways in which diabetes has been understood and its symptoms interpreted. Eight studies explored aspects of this relationship [10, 19–22, 26, 30, 32]. As we described earlier, most informants in literature we identified were unaware that their symptoms were indicative of diabetes, and instead attributed to them to other causes. Four studies found that initial interpretations of their symptoms lead many to assume that they had HIV/AIDS or other diseases such as malaria [8, 26, 27, 30]. This confusion of diagnosis was due to the lack of awareness of diabetes, the weight loss, inability to explain the cause of their symptoms, and the high rates of communicable diseases in their communities.

“Initially, I used to urinate a lot; also drank water a lot… When my condition further worsened and I continued to lose weight and couldn’t even go to the toilet by myself, I wondered if I might have contracted AIDS.” [26]

The assumption, in this Ethiopian context, that diabetes symptoms such as weight loss were attributable to HIV/Aids was not only held by those experiencing such symptoms them [10, 19–21, 26, 31]. Metta et al. describe how one participant described how other people thought that he had HIV/Aids [30], as did Awah, Unwin and Phillimore [10]. In such instances, participants were subject to similar forms of stigma as those living with HIV/Aids, as described by this Cameroonian:

“My weight was 90 kg when I was less than 35 years old. I have lost more than 10 kg as part of my treatment. People now perceive me as someone that is suffering from AIDS. This is affecting my personality. I have to gain some weight to remove that stigma and show them I am well...” [10]

One study found that participants had to continually explain their diagnosis, as family and community members thought that the person with diabetes was actually HIV positive.

“Why is your health so unstable… have you been tested (for HIV)?’ Even when you say I have been tested and I am just fine, you still hear: ‘You had better get another test at another place because sometimes they can miss it, if the second test shows you are ok, then you can be completely sure’” [30]

Stigma, as narrated by this Tanzanian person, also impacted the ways in which people with diabetes engaged with their treatment. From the four studies, paricipants detailed how they adjusted treatment regimens to avoid the stigma of the presumption of having HIV/AIDS. This was done by avoiding weight loss, or in the case of one Ghanian woman, ending treatment [19, 21].

## Discussion

This review found 21 studies relating to type 2 diabetes illness experiences in 8 of 48 SSA countries, published between 2003 and 2017. Interviews (mostly semi-structured) were the most common form of data collection (20/21) and focus groups were also popular (6/21). Studies in Ghana and South Africa accounted for nearly half of the records included in the review (10/21). At this very basic level of analysis, and as encouraged by scoping methodology [11], our work highlights a significant gap in knowledge relating to the illness experiences of people with type 2 diabetes in SSA. In plain terms: our searches returned no studies from 40/48 SSA countries.

With these geographical and cultural limitations in mind, we were nonetheless able to identify five areas of convergence in the literature from the 8 countries represented in the literature. Most studies (20/21) offered data on how people came to identify their diabetes, how they reacted to being diagnosed and how they located the condition using multiple pre-existing cultural frameworks. A majority of studies (13/21) described how biomedical, ethnomedical and faith treatments are often hybridised by people with diabetes in SSA, who locate their condition in multiple socio-cultural frameworks, are sometimes in pursuit of a cure, and often face financial and/or access issues that shape the treatment providers they can consult. All 21 studies included in the review reported on the various impediments people living in SSA face when pursuing care. These included issues of cost, access, interruptions to drug supplies and stress. Sources of support were described in 13/21 studies, with family and friends featuring heavily and some comment being made on levels of support received from medical professionals. Finally, intersection between HIV/AIDS and diabetes were described in 8/21 articles. Participants in these studies reported initially interpreting diabetes symptoms, such as weight loss, as signs of HIV/AIDS, and that this interpretation was often shared by others in their community, leading to stigma.

Taken together, the 21 studies paint living with type 2 diabetes in SSA as a complex and multifaceted experience, often characterised by considerable challenge. Our interpretation of the five themes synthesis that we have presented suggests that three major challenges can be identified in the literature. *Uncertainty* is arguably one of the leading challenges: supplies of medicine are interrupted [31]; incomes fluctuate and affect access to treatment [22]; visits to clinics can be disrupted by weather and cost [31]; and, above all, the condition itself can be difficult to anticipate and control [9]. Similarly, *economic resources* shape patient experiences: treatments (including travelling to obtain them) are unaffordable for many, especially when multiple drugs are prescribed [34]; diabetes limits earning potential, undermining resources available to fund care [22]; and resource constraints in health systems arguably limit the support that healthcare professional can provide patients with [9]. Finally, accessing *quality information and education* about diabetes is not easy in SSA: healthcare services are stretched, often leaving little time for education [33]; information is tailored to affluent patients, for example, through the assumption that access to medications is stable; and lack of information in communities contributes to the stigmatisation of diabetes, and to inabilities to identify the condition when confronted with symptoms [30].

While stemming from work in 8/48 countries, and with a limit on generalisability (10/21 were from 2 countries), these three challenges (*uncertainty, economic resource, and quality information/education*) are a useful starting point for focussed, collective action to improve the lives of those living with type 2 diabetes in SSA and could be addressed by a range of stakeholders. Policy makers could consider how they might reduce the uncertainties experienced by people with type 2 diabetes; how they might enhance the economic affordances offered to people with the condition; and how they may provide high-quality prevention and self-management education at both a population and localised (community) level. Health care professionals could develop explicit protocols for managing uncertainty, economic instability and access to quality information. Pharmaceutical companies could make greater efforts to ensure that cheap, first-line treatments such as metformin, are readily available. Finally, a range of civil society organisations, including national diabetes foundations, faith-based congregations/organisations, sports clubs, educational establishments and other socio-political collectives offer potential channels through which type 2 diabetes prevention and management strategies/programmes could be pursued.

A primary limitation of this scoping review is that it only admitted peer-reviewed articles from the databases that we searched. ‘Grey literature’ studies were omitted by design, which means that this review does not contain findings from non-peer reviewed studies, such as those produced by non-governmental organisations. This is a departure from the Arksey and O’Malley framework and resulted from time and resource constrains. However, the broad terms of our search offer the study a strength. Rather than narrowing our concern to specific aspects of the experiences of people with type 2 diabetes, our scoping review had no pre-conceived vision of what these experiences might be. An equally important limitation of the review is that it covers only 8/48 SSA territories, limiting generalisability. That said, this work has clearly identified a gap in knowledge that needs redressing if SSA countries are to improve the illness experiences of those with type 2 diabetes. Limiting our searches to studies in English is a further limitation, but one that was unavoidable due to the language skills present in the team. Finally, our consultations could have been broader, which may have resulted in additional literature being identified.

## Conclusions

Research focussed on exploring the illness experiences of people living with type 2 diabetes is yet to cover the broad territory that is SSA. Based on an analytical synthesis of the evidence presented in 21 studies from 8/48 SSA countries, this scoping review has identified *uncertainties*, *economic resources* and *quality information and education* as key concerns for people living with diabetes in SSA. Governments, civil society organisations, culturally-sensitised public health practitioners and diabetes specialists could work collaboratively to produce interdisciplinary and pragmatic approaches to management and care for those living with type 2 diabetes, as well as prevention for those who are at risk of developing the condition.

## Abbreviations

NCDs: Non-communicable diseases
SSA: Sub-Saharan Africa
USD: United States Dollars
